# Positional Relationship Between the Orbicularis Oculi and Zygomaticus Complex Muscles by Ultrasonography: New Anatomical Insights for Crow's Feet Injection

**DOI:** 10.1002/ca.24290

**Published:** 2025-05-07

**Authors:** Jiong‐Zhen Piao, Ji‐Hyun Lee, Kyung‐Seok Hu, Hee‐Jin Kim

**Affiliations:** ^1^ Division in Anatomy and Developmental Biology, Department of Oral Biology, Human Identification Research Institute, BK21 FOUR Project Yonsei University College of Dentistry Seoul Korea; ^2^ Department of Anatomy and Acupoint, College of Korean Medicine Gachon University Seongnam Korea; ^3^ Department of Electric and Electronical Engineering, College of Engineering Yonsei University Seoul Korea

**Keywords:** botulinum toxin, crow's feet, lateral canthal lines, orbicularis oculi, ultrasonography, zygomaticus major, zygomaticus minor

## Abstract

The orbicularis oculi muscle (OOc) is strongly associated with facial aging as its contraction causes the formation of crow's feet. Botulinum neurotoxin (BoNT) injection is a representative treatment targeting muscle. The aim of this study was to demonstrate the anatomical relationship between the OOc and the zygomaticus complex muscles, and to visualize the distribution of the facial nerve that innervates the OOc, thereby providing reference data for BoNT injections targeting that muscle. The positional relationships and overlapping ranges between the OOc, zygomaticus minor (Zmi), and zygomaticus major (ZMj), and their distances from the skin, were measured on four different perpendicular planes using ultrasonography. Specimens of the OOc were stained with modified Sihler's stain. The mean distances between the lateral canthus horizontal plane (LCHP) and the zygomaticus complex muscles superior margin were 20.0, 17.9, 22.8, and 20.8 mm in perpendicular planes LC (lateral canthus), OR (orbital rim), M (midpoint of the frontal process of zygomatic bone), and J (Jugale point), respectively. The mean distances between the OOc and the skin were 4.9, 4.8, 5.5, and 4.7 mm in those perpendicular planes. The mean distances between the zygomaticus complex muscles and the OOc were 3.0, 3.1, 4.5, and 4.1 mm. The authors propose new insights for crow's feet injection based on anatomical information obtained from ultrasonography and Sihler's staining, which should contribute to minimizing complications and improving the efficacy of BoNT administration.

## 1 Introduction

The orbicularis oculi (OOc) is an elliptically broad and flattened muscle involved in a range of facial expressions (Kim et al. 2021). This involvement stems from its overlaps with neighboring muscles to which it is directly or indirectly connected (Park et al. 2011). The OOc consists of three parts, delineated on the basis of their location: pretarsal, preseptal, and orbital. The orbital part is strongly associated with facial aging as its contraction causes the formation of crow's feet, otherwise known as lateral canthal lines. Previous studies suggest that the point at which botulinum neurotoxin (BoNT) is injected into the OOc for crow's feet should be 1.5–2.0 cm lateral to the lateral canthus (LC) (Kim et al. 2016).

Injecting BoNT into the lateral and lower aspects of the OOc and its most lateral portion is not recommended. The former can weaken the zygomaticus major (ZMj) muscle, leading to an inability to elevate the corners of the mouth. The latter considers the possibility of anatomical variations of the OOc, which could weaken its lateral muscular band and lead to an asymmetrical unnatural smile (Park et al. 2011; Seo 2017). The positional relationships between the OOc, zygomaticus minor (Zmi), and ZMj are the main determinants of the precise injection point. These considerations are summarized in the guidelines for OOc BoNT injection and cosmetic surgery (Park et al. 2011; Youn et al. 2012; Choi et al. 2014; Hur et al. 2018; Kampan et al. 2017). The Zmi and ZMj function to elevate the upper lip and the corners of the mouth and are jointly known as the zygomaticus complex muscles.

However, previous research on the anatomical relationships between the OOc, Zmi, and ZMj has been conducted using fixed cadavers, most of which were from aged donors. It is well known that the topography of the facial muscles differs significantly between aged and young individuals. Moreover, previous research focused on classifying the positional relationship between the OOc and the zygomaticus complex muscles, but data contributing to clinical applications were lacking.

Ultrasonography has recently been used to examine the craniofacial area in order to target small muscles or determine precise injection points; it can identify muscle and soft tissue layers in real time (Kim et al. 2021; Bae et al. 2020; Ahn et al. 2020; Lee et al. 2023). Sihler's staining can be used to reveal the density, distribution, and pathways of nerves in the facial area, thereby providing an effective guideline for BoNT injections (Yang et al. 2013).

The aim of this study was to assess the anatomical relationship between the OOc and the zygomaticus complex muscles, and to visualize the distribution of the facial nerve that innervates the OOc, thereby providing reference data for BoNT injections targeting crow's feet and for periorbital cosmetic surgery.

## 2 Materials and Methods

### 2.1 Ultrasonographic Examination

The study was conducted in accordance with the guidelines of the Declaration of Helsinki and was approved by the Institutional Review Board of the institution overseeing the research (IRB approval number: Redacted for Peer Review). The cadavers used were legally donated to institutes. The orbital regions were dissected after approval from the institution overseeing this research. The subjects had provided consent for donating their bodies for research purposes. The authors state that every effort was made to follow all local and international ethical guidelines and laws that pertain to the use of human cadaveric donors in anatomical research (Iwanaga et al. 2022a).

**FIGURE 1:**
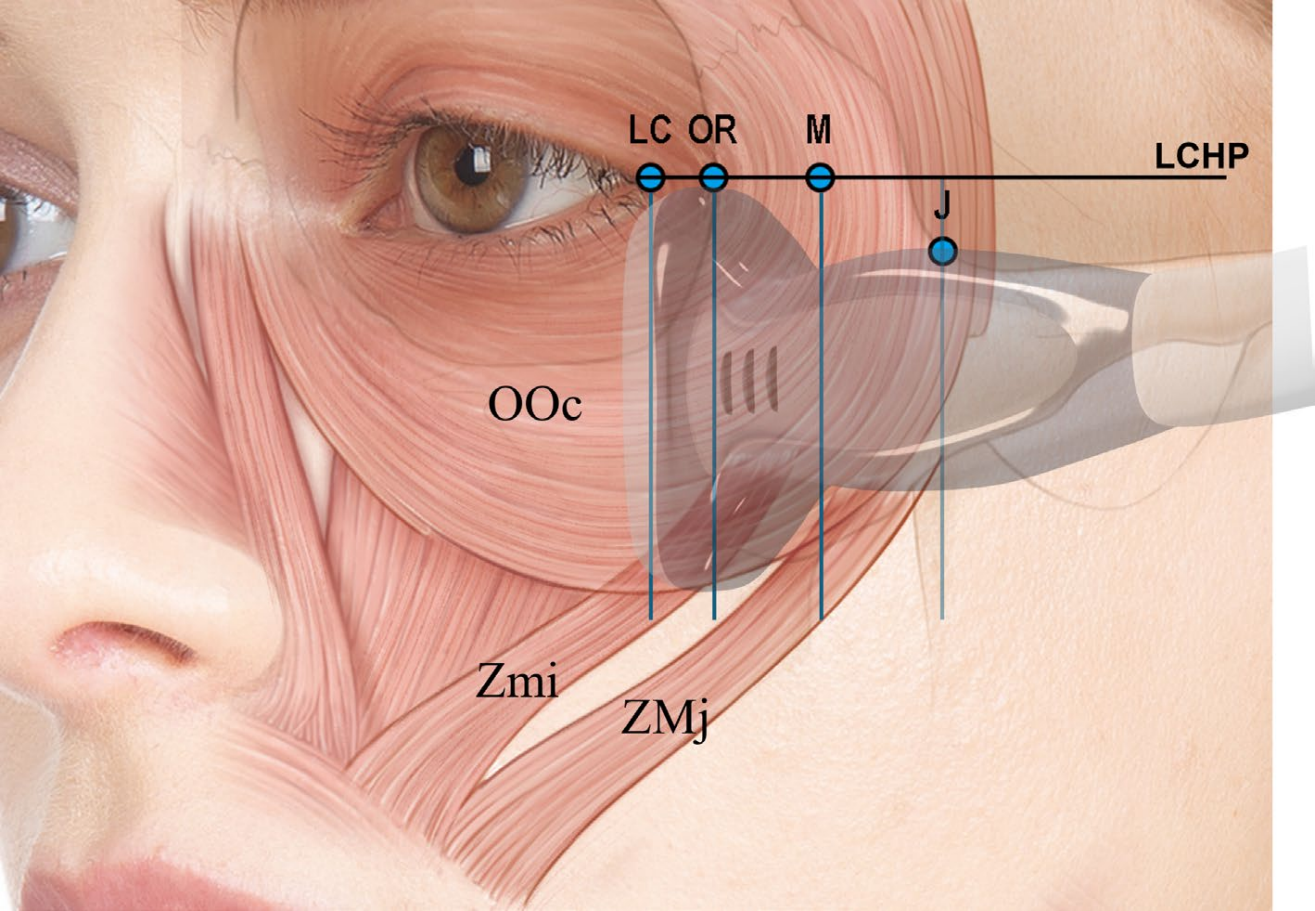
Illustration representing reference lines for ultrasonographic examination of the orbicularis oculi muscle and zygomaticus complex muscles. A horizontal line was drawn laterally from the lateral canthus (LC), and the 3 landmarks were marked where this line intersected with the LC, OR and M point. The J referred to jugale point. There were 4 perpendicular lines passing through the LC, OR, M and J. J, jugale point; LCHP, lateral canthus horizontal plane; M, the midpoint of the frontal process of zygomatic bone; OOc, orbicularis oculi muscle; OR, orbital rim; Zmi, zygomaticus minor muscle; ZMj, zygomaticus major muscle.

**FIGURE 2:**
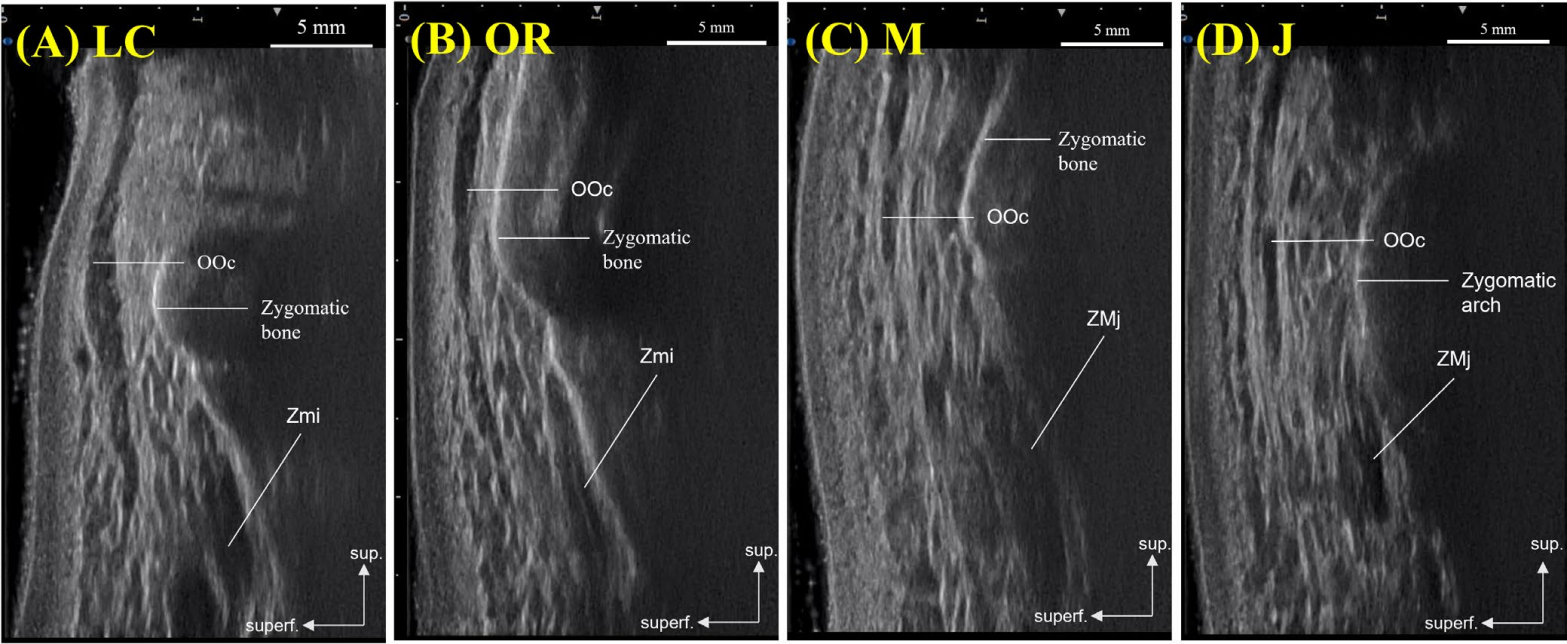
Ultrasonographic examination of the orbicularis oculi muscle and the zygomaticus complex muscle. (A) B mode image of the OOc and zygomaticus minor muscle (Zmi) at the perpendicular plane LC. (B) B mode image of the OOc and Zmi at the perpendicular plane OR. (C) B mode image of the OOc and zygomaticus major muscle (ZMj) at the perpendicular plane M. (D) B mode image of the OOc and ZMj at the perpendicular plane J. J, jugale point; LC, lateral canthus; M, the midpoint of the frontal process of zygomatic bone; OR, orbital rim.

**FIGURE 3:**
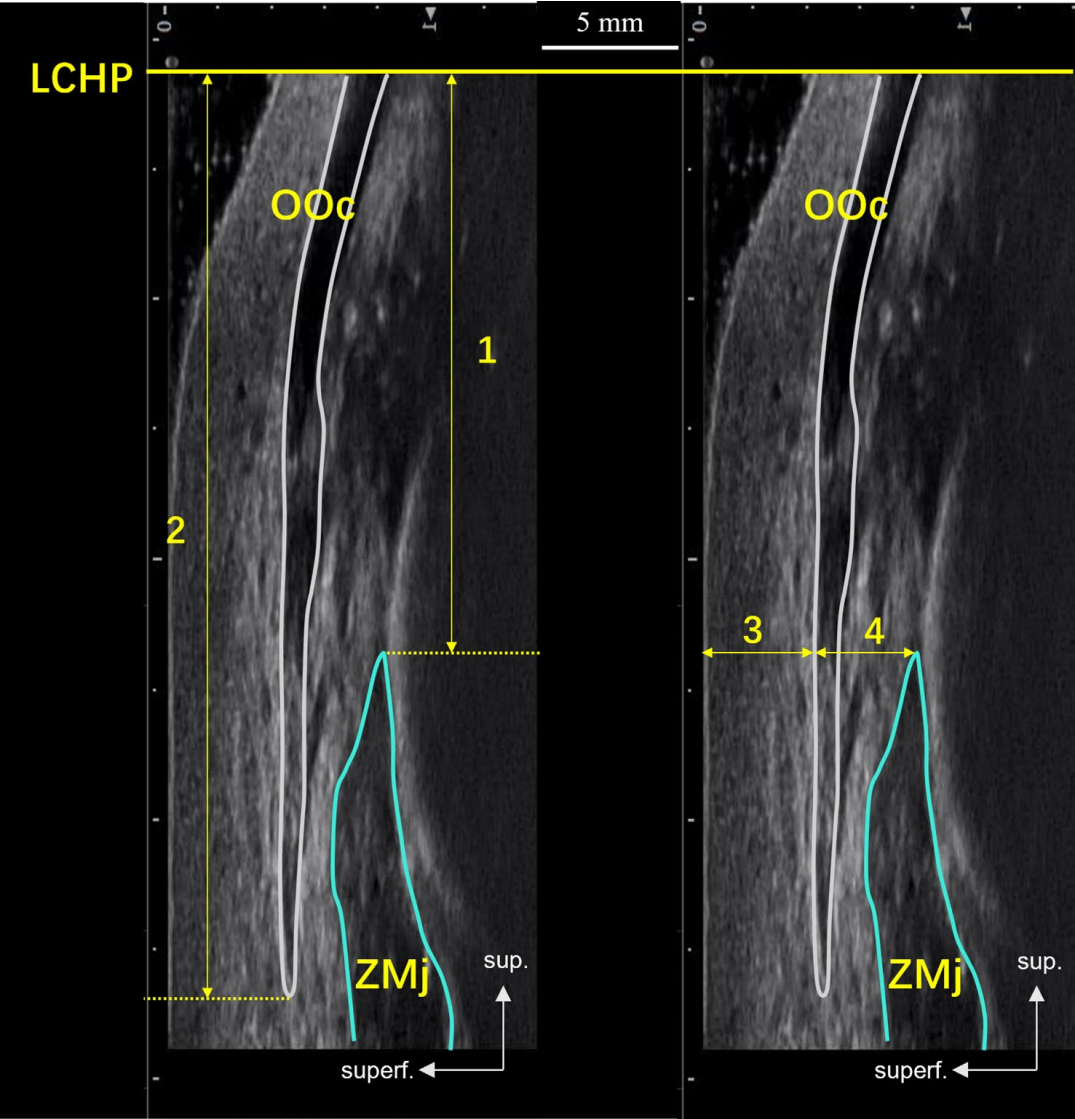
The 4 parameters measured on ultrasonographic examination of the orbicularis oculi muscle and the zygomaticus complex muscles. 1, the distance from the superior margin of the zygomaticus complex muscles to the lateral canthus horizontal plane (LCHP); 2, the distance from the inferior margin of the orbicularis oculi muscle (OOc) to the LCHP; 3, the distance from the epidermis to the surface of the OOc in the horizontal plane of the superior margin of the zygomaticus complex muscles; 4, the distance from the OOc to the surface of the zygomaticus complex muscles in the horizontal plane of the superior margin of the zygomaticus complex muscles. ZMj, zygomaticus major muscle.

The volunteers were positioned semi‐supine for ultrasonography. The positional relationship and overlapping ranges between the OOc, Zmi, and ZMj, and their distances from the skin, were measured on four perpendicular reference lines using a two‐dimensional B‐mode ultrasound device with a high‐frequency (16 MHz) linear transducer (Sonimage HS1, KONICA MINOLTA, Tokyo, Japan). A horizontal line was drawn laterally from the LC, and landmarks were indicated where this line intersected with the LC, orbital rim (OR), and the midpoint of the frontal process of the zygomatic bone (M) (Figure 1). The jugale point (J) was confirmed by palpation. Four perpendicular lines passing through the LC, OR, M, and J were established. Ultrasonography was performed at these lines by aligning the edge of the transducer with the lateral canthus horizontal plane (LCHP) (Figure 1). In each perpendicular plane, the superficially located OOc and the deeply located bony origins of the Zmi and ZMj could be identified (Figure 2). We measured four parameters on each reference line. The distance from the LCHP to the superior margin of the Zmi and ZMj, and to the inferior margin of the OOc was measured. The depth of these muscles was measured in the horizontal plane of the superior margin of the Zmi and ZMj (Figure 3). The positional relationships and overlapping ranges of the OOc, Zmi, and ZMj, in conjunction with the depths of these muscles, were measured using Image J (National Institutes of Health, Bethesda, MD, USA). Forty hemiface data were collected from 20 healthy South Korean volunteers (seven men and 13 women; mean age, 28.9 years) with no history of facial cosmetic procedures during the previous 6 months.

### 2.2 Sihler's Staining

**FIGURE 4:**
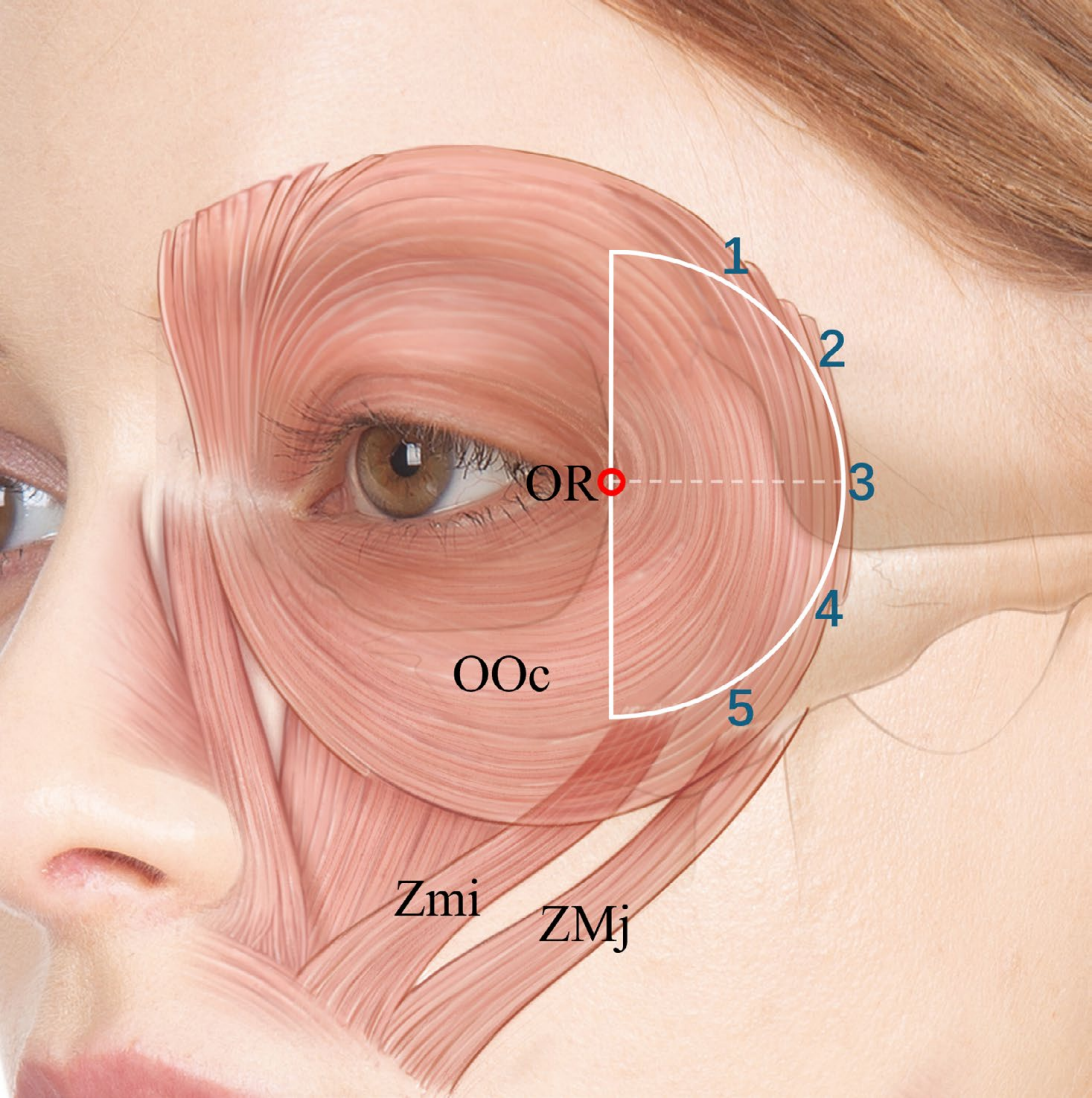
A schematic semicircular model of the orbicularis oculi muscle for Sihler's staining. A schematic representation of the orbicularis oculi muscle (OOc) was delineated as a semicircular model using the orbital rim (OR) point as the center and the distance from the OR to the lateral margin of the OOc at the lateral canthus horizontal plane as the radius. Zmi, zygomaticus minor muscle; ZMj, zygomaticus major muscle.

A total of 11 OOc specimens obtained from frozen cadavers of donors with no history of facial trauma or plastic surgery were dissected. Sihler's staining seriously alters the morphology of the tissue, which can undergo significant shrinkage and distortion. Therefore, to present the facial nerve topography in the stained specimens accurately, we marked the bony landmark by tying knots prior to removing the specimen, ensuring the provision of easily identifiable and less anatomically variable landmarks. Construction of a semicircular arc using the designated OR point as the center, and the distance from the OR to the lateral margin of the OOc at the LCHP as the radius, ensured accurate representation of the nerve distribution after staining (Figure 4). These cadavers were meticulously dissected, and the OOc were isolated together with neighboring soft tissues. Sihler's staining was implemented on each specimen as follows:

Fixation: The dissected specimens were fixed in 10% un‐neutralized formalin for 14 days, the solution being replaced when cloudy.

Maceration and depigmentation: The fixed specimens were washed under running tap water for 60 min and then placed for 4 weeks in 3% aqueous potassium hydroxide with 0.2 mL of 3% hydrogen peroxide per 100 mL; the solution was changed every day.

Decalcification: The macerated specimens were washed under running tap water for 60 min. They were placed in Sihler's solution I (1:1:6 glacial acetic acid, glycerol, and 1% aqueous chloral hydrate) for 3 weeks; the solution was changed once a week.

Staining: The decalcified specimens were colorized with Sihler's solution II (1:1:6 Ehrlich hematoxylin, glycerol, and 1% aqueous chloral hydrate) for 3–4 weeks.

Destaining: The stained specimens were washed for 60 min and then placed in Sihler's solution I; the solution was changed whenever it turned purple. The process was terminated when the stained nerve fibers started to exhibit signs of fading.

Neutralization: The destained specimens were washed for 60 min and then placed in a 0.05% lithium carbonate solution for 1 h.

Cleaning: The specimens were placed in a series of increasing concentrations of glycerol (from 40% to 100%, the concentration being increased by 20% every day).

## 3 Results

### 3.1 The Locations of the Zmi, ZMj Superior Margin and OOc Inferior Margin at Each Perpendicular Plane

**FIGURE 5:**
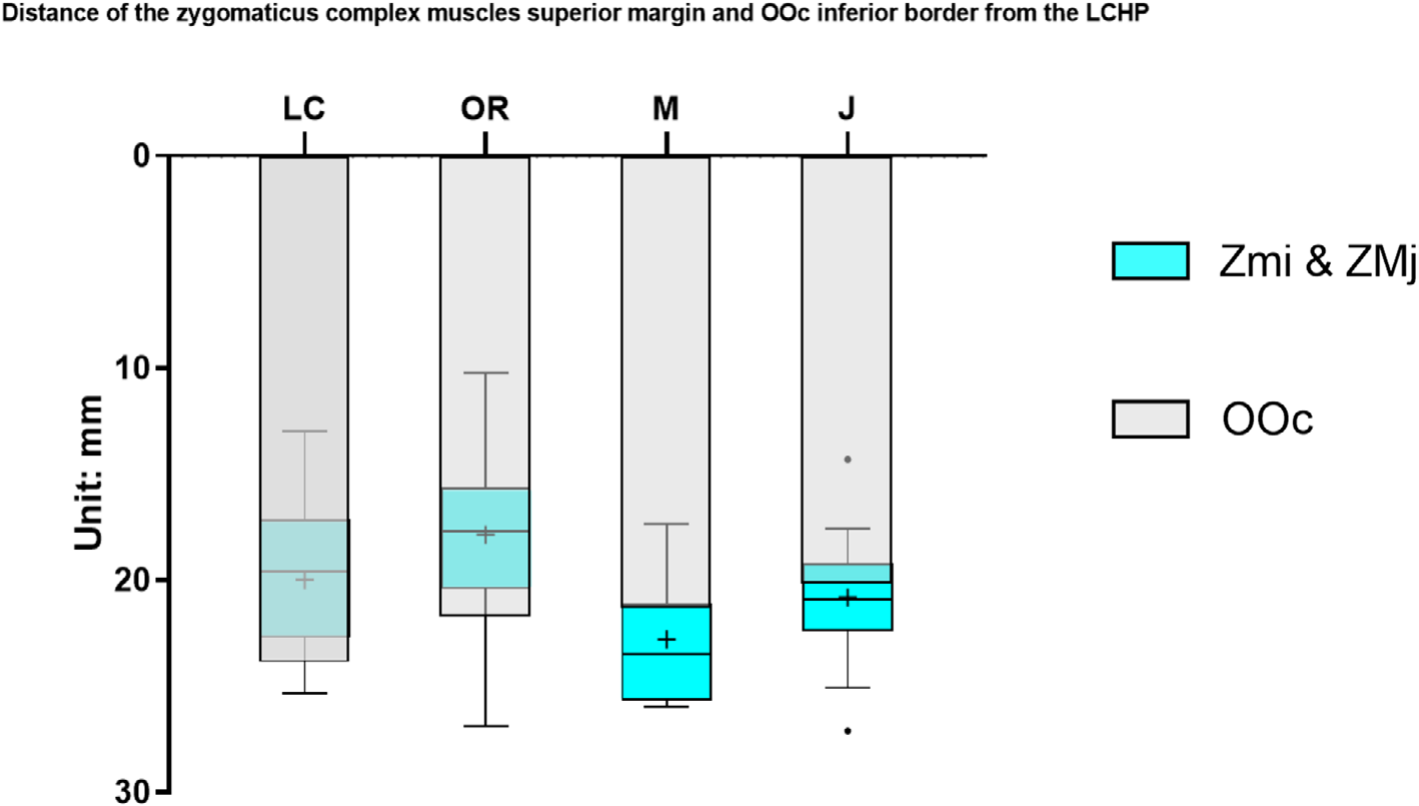
Distance of the zygomaticus complex muscles superior margin and orbicularis oculi muscle inferior margin from the lateral canthus horizontal plane. LC‐Box plot, the distance from the Zmi & ZMj superior margin to the lateral canthus horizontal plane (LCHP) at LC line. OR‐Box plot, the distance from the Zmi & ZMj superior margin to the LCHP at OR line. M‐Box plot, the distance from the Zmi & ZMj superior margin to the LCHP at M line. J‐Box plot, the distance from the Zmi & ZMj superior margin to the LCHP at J line. LC‐Bar chart, the distance from the OOc inferior margin to the LCHP at LC line. OR‐Bar chart, the distance from the OOc inferior margin to the LCHP at OR line. M‐Bar chart, the distance from the OOc inferior margin to the LCHP at M line. J‐Bar chart, the distance from the OOc inferior margin to the LCHP at J line. J, jugale point; LC, lateral canthus; M, the midpoint of the frontal process of zygomatic bone; OOc, orbicularis oculi muscle; OR, orbital rim; Zmi, zygomaticus minor muscle; ZMj, zygomaticus major muscle.

The distances of the Zmi, ZMj superior margin, and OOc inferior margin from the LCHP at each perpendicular plane are presented in Table 1 and Figure 5. The mean distances between the LCHP and the superior margin of the zygomaticus complex muscles were 20.0 mm (13.0–25.1 mm), 17.9 mm (10.2–26.9 mm), 22.8 mm (17.4–26.0 mm), and 20.8 mm (14.3–27.1 mm) at perpendicular plane LC, OR, M, and J, respectively. The mean distances between the LCHP and the OOc inferior margin were 23.8 mm (18.0–32.5 mm), 21.7 mm (16.3–29.2 mm), 21.3 mm (17.2–25.8 mm), and 20.1 mm (15.6–28.3 mm) at perpendicular plane LR, OR, M, and J, respectively. The locations of the Zmi and ZMj were more variable at perpendicular plane OR than the others; the location of the OOc was most variable at perpendicular plane LC. There were no statistically significant sex or side differences (*p* > 0.05).

**TABLE 1:** Distance of the zygomaticus complex muscles superior margin and orbicularis oculi muscle inferior margin from the lateral canthus horizontal plane.

	LC	OR	M	J
Zmi & ZMj				
Mean ± SD	20.0 ± 3.6	17.9 ± 3.9	22.8 ± 2.9	20.8 ± 2.7
Minimum	13.0	10.2	17.4	14.3
Maximum	25.1	26.9	26	27.1
OOc				
Mean ± SD	23.8 ± 4.1	21.7 ± 3.6	21.3 ± 2.8	20.1 ± 3.1
Minimum	18.0	16.3	17.2	15.6
Maximum	32.5	29.2	25.8	28.3

*Note: N* = 40, unit: mm.

Abbreviations: J, jugale point; LC, lateral canthus; M, the midpoint of the frontal process of zygomatic bone; OOc, orbicularis oculi muscle; OR, orbital rim; Zmi, zygomaticus minor muscle; ZMj, zygomaticus major muscle.

### 3.2 Depth of the Zmi, ZMj, and OOc at Each Perpendicular Plane

**FIGURE 6:**
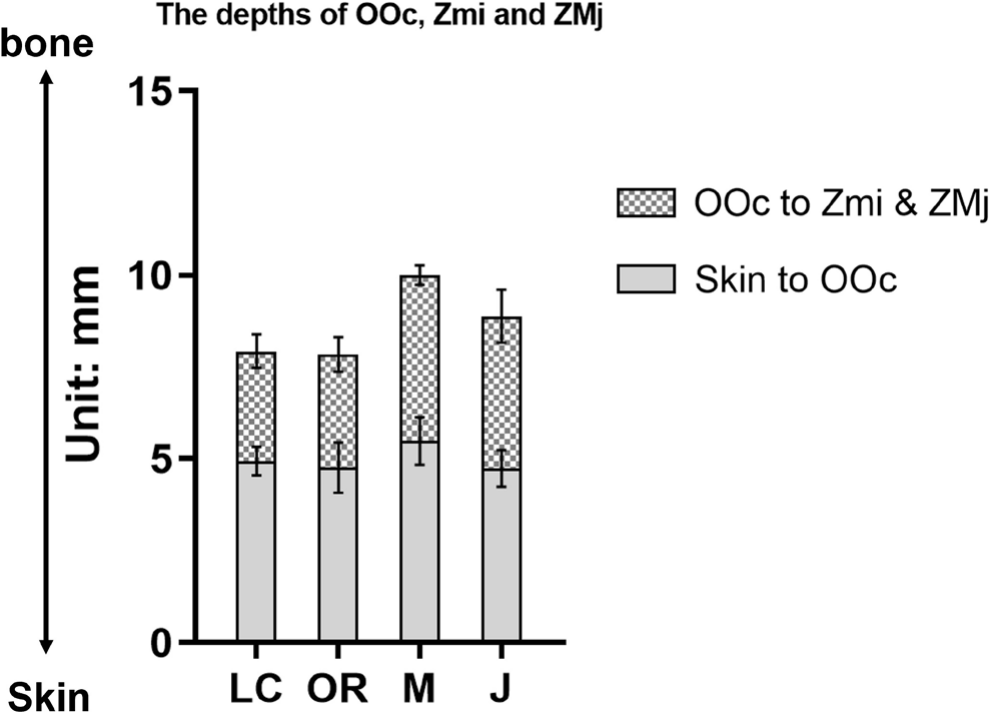
Depths of the orbicularis oculi muscle and the zygomaticus complex muscles in the horizontal plane of the superior margin of the zygomaticus complex muscles. The gray diagram indicates the distance from the skin to the OOc includes the thickness of the skin, subcutaneous tissue. The dotted diagram indicates the distance from the OOc to the Zmi & ZMj. J, jugale point; LC, lateral canthus; M, the midpoint of the frontal process of zygomatic bone; OOc, orbicularis oculi muscle; OR, orbital rim; Zmi, zygomaticus minor muscle; ZMj: zygomaticus major muscle.

We measured the depth from the skin to the OOc and from the OOc to the zygomaticus complex muscles in the horizontal plane of the superior margin of the zygomaticus complex muscles (Figure 6 and Table 2). The mean distances between the OOc and the skin were 4.9 mm (3.2–6.4 mm), 4.8 mm (2.6–6.7 mm), 5.5 mm (3.4–7.8 mm), and 4.7 mm (2.9–6.4 mm) at perpendicular plane LR, OR, M, and J, respectively. The mean distances between the zygomaticus complex muscles and the OOc were 3.0 mm (1.5–4.4 mm), 3.1 mm (1.5–4.9 mm), 4.5 mm (3.1–5.3 mm), and 4.1 mm (2.5–6.4 mm) at perpendicular plane LR, OR, M, and J, respectively. There were no statistically significant sex or side differences (*p* > 0.05).

**TABLE 2:** Depths of the orbicularis oculi muscle and the zygomaticus complex muscles in the horizontal plane of the superior margin of the zygomaticus complex muscles.

	LC	OR	M	J
Skin to OOc				
Mean ± SD	4.9 ± 0.4	4.8 ± 0.7	5.5 ± 0.6	4.7 ± 0.5
Minimum	3.2	2.6	3.4	2.9
Maximum	6.4	6.7	7.8	6.4
OOc to Zmi & ZMj				
Mean ± SD	3.0 ± 0.5	3.1 ± 0.5	4.5 ± 0.3	4.1 ± 0.7
Minimum	1.5	1.5	3.1	2.5
Maximum	4.4	4.9	5.3	6.4

*Note: N* = 40, unit: mm.

Abbreviations: J, jugale point; LC, lateral canthus; M, the midpoint of the frontal process of the zygomatic bone; OOc, orbicularis oculi muscle; OR, orbital rim; Zmi, zygomaticus minor muscle; ZMj, zygomaticus major muscle.

### 3.3 Sihler's Staining

After the staining, semi‐transparent OOc and transparent soft tissues were obtained. This made it easier to visualize the facial nerve distribution within the OOc and the nerve pathways in the soft tissues, discernible by the naked eye up to the fine terminals.

**FIGURE 7:**
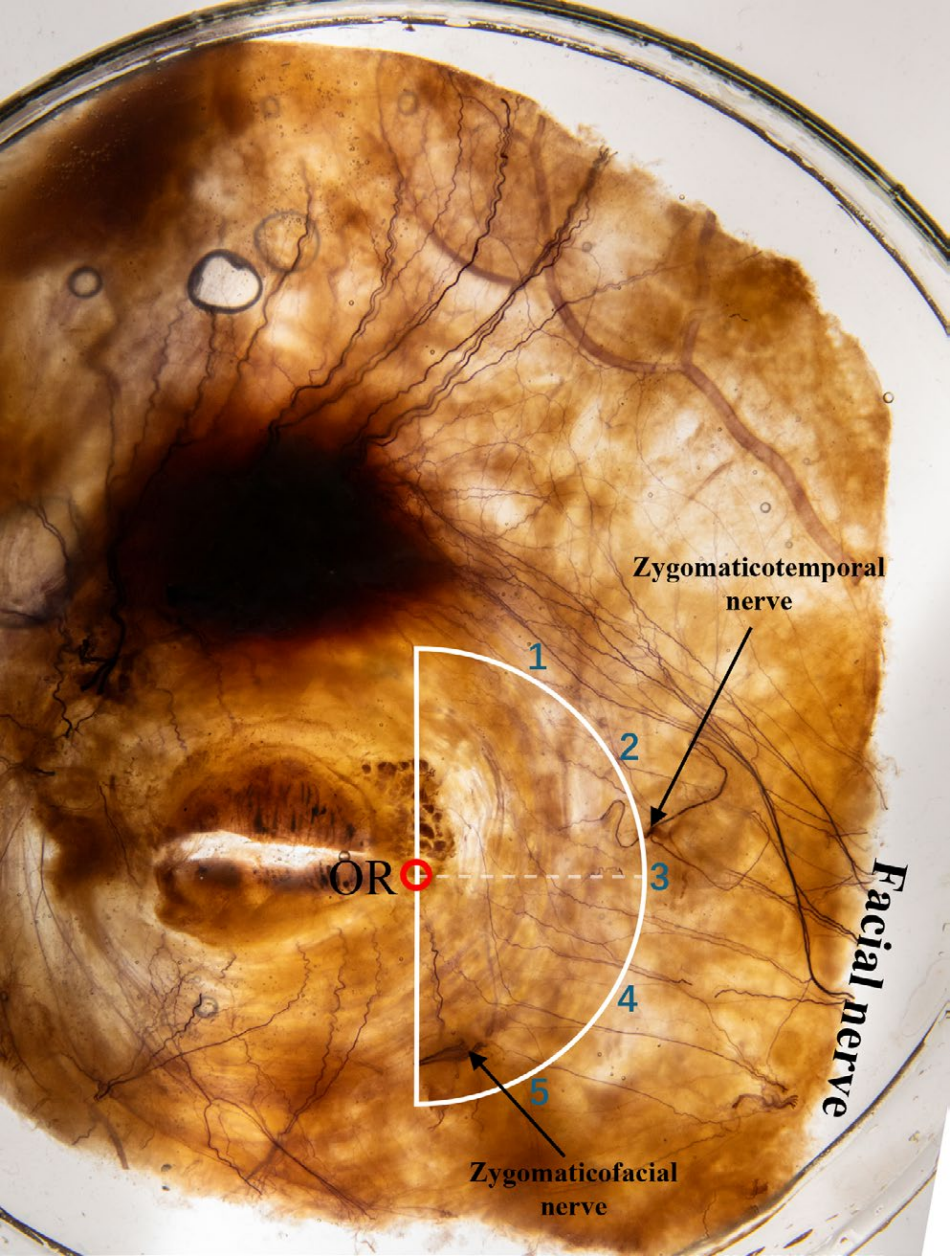
Facial nerve distribution within the orbicularis oculi muscle. OR, the point at the orbital rim.

**FIGURE 8:**
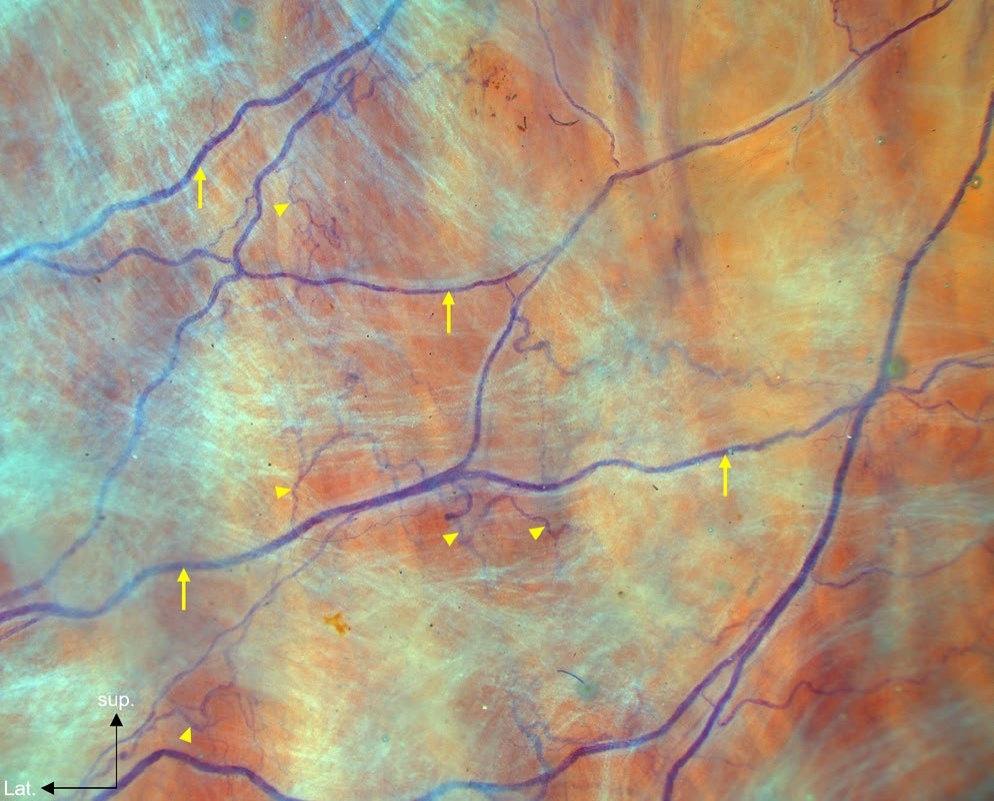
Posterior view of the orbicularis oculi muscle (OOc) with Sihler's staining. This image corresponds to the enlarged OOc at 10× magnification. The facial nerve (arrow, purple staining) travels beneath OOc and branches out into fine branches (arrowhead) to innervate the OOc located superficially.

The nerve branches converging laterally toward the muscle tissue were densely distributed in the 1–4 o'clock range of the semicircular model (Figure 7). It is evident from the diagram that this region is appropriate for injection owing to its rich nerve distribution. The nerve distribution revealed by Sihler's staining appears consistent with that seen in previous gross anatomical investigations (Yang et al. 2013; Choi and Kim 2020). However, we found during stereomicroscopy (Leica MZ FLIII, Heerbrugg, Switzerland; 10× magnification) that the facial nerve does not travel within the OOc, but through the soft tissue beneath it. Stereomicroscopy showed that the distribution of the facial nerve does not follow a pattern in which some nerves terminate at the orbital part and others at the palpebral part. Instead, the facial nerve fibers travel continuously through the orbital part without terminating. As the facial nerve progresses, it forms fine branches to innervate the orbital part of the OOc, which is located superficially (Figure 8).

## 4 Discussion

The aim of this study was to establish anatomical evidence to facilitate precise crow's feet treatment and periorbital cosmetic surgery by elucidating the positional relationships between the OOc and the zygomaticus complex muscles, and visualizing the distribution of the facial nerve within the OOc musculature.

BoNT injection remains the most prevalent cosmetic procedure for addressing facial wrinkles. In view of its minimal side effects, effective outcomes, and high patient satisfaction, it has been applied extensively to crow's feet (Breidenbach and Brunger 2005; Sundaram et al. 2016; Small 2014; Hexsel et al. 2011). However, some associated complications have been reported. As the toxin infiltrates not only the targeted OOc but also the adjacent Zmi and ZMj, patients can exhibit symptoms of Bell's palsy (Matilde and Spósito 2002; Spiegel and DeRosa 2005). This emphasizes the need for precise targeting of the injections. If clinicians inadvertently inject the toxin into the overlapping Zmi or ZMj, patients can experience muscle weakness, resulting in an inability to elevate the corners of the mouth and upper lip. Understanding these anatomical relationships is paramount to avoiding the risk of adverse complications.

In this study, we determined the positional relationships between the OOc and zygomaticus complex muscles on four perpendicular planes. In contrast to previous studies, we determined the overlapping ranges and depth of the OOc and the zygomatic complex muscles by ultrasonography of young individuals and performed Sihler's staining on fresh cadavers. Our study provides clinical anatomical information that can help practitioners target the BoNT injection more precisely.

We investigated the bony origins of the Zmi and ZMj along each perpendicular plane using sagittal ultrasound images of the lateral part of the OOc. Considering the average distance between the superior margin of the Zmi and ZMj and the inferior margin of the OOc from the LCHP on the four perpendicular planes can allow us to avoid injections into the overlapping region. Because BoNT diffuses during injection, exceeding the depth of the OOc significantly increases the risk of its spreading to the Zmi and ZMj. The ultrasound images make it evident that the subcutaneous tissue gradually thickens from the superior to the inferior aspect, indicating an increase in muscle depth. This is consistent with a previous study that used a three‐dimensional scanning system to determine the thickness of the facial skin and subcutaneous tissue (Kim et al. 2019). We therefore measured the muscle depth in the horizontal plane at the superior margin of the Zmi or ZMj. Using this depth as a reference makes it feasible to avoid injections into the Zmi and ZMj, even in the overlapping region. Additionally, as the dosage and dilution of BoNT influence the field of effect, those factors should be considered in clinical practice (Ramirez‐Castaneda et al. 2013). Previous studies indicated that the diffusion of BoNT into adjacent muscles is dose‐dependent, and multiple injection points with small doses can effectively reduce the extent of toxin diffusion (Borodic et al. 1994; Shaari et al. 1991). It has also been reported that high dilution of administered BoNT can result in wider diffusion and a larger affected area (Hsu et al. 2004; Kranz et al. 2010). However, there is insufficient evidence to confirm whether varying dilution ratios can reliably predict the diffusion effect. Since the extent of the denervated area is mainly influenced by toxin dose and volume, performing multi‐points rather than a single bolus injection should limit the effects of the toxin to the targeted muscle.

We aimed to minimize the risk of OOc injection for crow's feet treatment by avoiding the lip elevator and mouth corner elevator muscles. Given the overlapping of the muscles, it is crucial not only to avoid injecting into the overlapping regions, but also to consider the depth of the injection carefully. Ultrasonographic visualization helps to determine the optimal injection method and dosage by providing precise insights into the location and layer of the target muscle (Lee et al. 2018). Moreover, being non‐invasive, non‐radioactive, and able to reveal anatomical variations, it enables facial muscle topography to be assessed in young living subjects. The anatomical information acquired from young individuals is more appropriate for clinical applications than that acquired from fixed cadavers.

One limitation of this study is that the Zmi and ZMj we investigated were of bony origin, whereas in reality they consist of fibers originating from both bone and the OOc. Hur et al. reported that OOc fibers were involved in the composition of the Zmi in all specimens (Hur et al. 2018). Kampan et al. reported that the lateral bundles of the OOc joined with the ZMj in all specimens, and with the Zmi in 63.6% of 12 cadavers of Japanese individuals (Kampan et al. 2017). Another recent study suggests that the OOc and ZMj are connected in only 22.7% (Iwanaga et al. 2022b). In contrast to typical skeletal muscles, the facial expression muscles lack a distinct fascial envelope, and their thin and flat nature makes it challenging to identify merged muscles in ultrasound images. Therefore, ultrasonography cannot differentiate between muscle bundles originating from the zygomatic bone and those extending from the OOc.

The origin of the Zmi and ZMj varies among ethnic groups. Data obtained from studies on African, Hawaiian, and Chinese populations indicate that OOc are commonly connected to the undifferentiated zygomaticus muscle groups. In contrast, in most White subjects, the zygomaticus musculature is independent of the OOc, with no connection between them.

Both the Zmi and ZMj originate from the zygomatic arch (Ernst 1932). Given that some of the fibers originate from the OOc, OOc toxin injection anatomically affects the zygomaticus complex muscles, and therefore upper lip and mouth corner movements. Nonetheless, this anatomical relationship has been explored in several recent studies. A previous study demonstrated that the anatomically variant lateral muscular band of OOc terminates at the corner of the mouth, and assessed the position of this band at the level of the LC (Park et al. 2011). Hur et al. suggested that the mean (± SD) width of the lateral fibers of the OOc that extended to the upper lip at the LC level was 6.9 (± 3.3 mm) (Hur et al. 2023). These data provide guidance for avoiding injecting into the OOc fibers that extend to the zygomaticus complex muscles. Another previous study attributed the formation of lower crow's feet and lateral cheek rhytides, referred to as accordion wrinkles, to the simultaneous contraction of the OOc and ZMj (Mole 2013). Thus, although anatomical information indicates that injecting into the connecting fibers of the OOc can affect the ZMj, such injection can help to reduce accordion wrinkles. Further investigation is necessary to re‐evaluate the effects of such injection on facial wrinkles and the balance of action.

Visualization of the nerve in the orbital region can clarify the injection guidelines for crow's feet. Clinical, pharmacological, and anatomical factors are critical determinants of the efficacy and safety of BoNT injections. The technical approach to achieving effective injection is concerned with the efficiency of agents that approach the intramuscular motor end plates (Cho et al. 2023). Therefore, determining the nerve density within the targeted anatomical region is critical for ensuring the optimal efficacy of BoNT administration (Warden et al. 2014; Li et al. 2021; Wang et al. 2022). Several studies have used conventional gross anatomical methods and revealed a nerve distribution in the OOc similar to that reported in the present study (Ramirez and Santamarina 2000; Hwang et al. 2004). As Sihler's staining delineates nerve distribution with enhanced detail, it should be taken into consideration for BoNT injection.

According to the BoNT injection guidelines for typical skeletal musculature, the optimal injection points can be determined on the basis of the abundance of neuromuscular junctions (Lee et al. 2022). A previous study used microdissection and Sihler's staining to determine the arborization of the masseteric nerve branches within the masseter muscle and proposed that injecting into the area with richest arborization facilitates the treatment of masseteric hypertrophy (Kim et al. 2010). As BoNT acts on nerve endings, anatomical knowledge of the location of the nerve endings in the target muscle is crucial for optimal efficacy with minimum BoNT dosage (Lee et al. 2020). Nevertheless, unlike the masseteric nerve, which branches off twigs within the masseter muscle and innervates its superficial, middle, and deep layers, stereomicroscopy showed a distinct pattern for the facial nerve in the OOc.

Stereomicroscopic magnification helps to visualize specimens three‐dimensionally, thereby allowing a thorough examination to be performed. It shows that the facial nerve does not enter directly into the flattened OOc to branch off twigs, but branches off superficially to the OOc as it progresses medially through the soft tissue beneath that muscle. Another study using histological staining of coronal sections along the reference line perpendicular to the jugale point revealed the facial nerve in the perpendicular plane, located within the soft tissue layer deep to the OOc (Lee et al. 2023). Hence, the limitation of Sihler's staining lies in its difficulty in identifying precise injection points on the basis of the richest distribution of nerve endings in the OOc; the nerve distribution seen in this study is shown as the pathway of the facial nerve beneath the OOc. Considering that, the OOc itself is small, and that toxin diffusion has a certain range, we recommend injection points based on the areas in which nerve pathways were relatively concentrated in the stained specimen. Furthermore, precise anatomical information is of greater clinical significance than neuromuscular junction abundance.

## 5 Conclusion

In this study, we propose new insights for crow's feet injection based on anatomical information obtained from ultrasonography and Sihler's staining. Ultrasonography was used to quantify the distances between the superior margin of the zygomaticus complex muscles and the inferior margin of the OOc from the LCHP on four perpendicular planes. In clinical practice, it is important that ultrasonographical examination reveals the anatomical variations among individuals in real time. Therefore, the results of this study should contribute to minimizing complications during the administration of BoNT for treating crow's feet.

## Ethics Statement

The study was conducted in accordance with the guidelines of the Declaration of Helsinki and was approved by the Institutional Review Board of Yonsei University Health System, Severance Hospital (IRB no. 2‐2023‐0050).

## Data Availability

The data that support the findings of this study are available from the corresponding author upon reasonable request.
